# Drug safety assessment in clinical trials: methodological challenges and opportunities

**DOI:** 10.1186/1745-6215-13-138

**Published:** 2012-08-20

**Authors:** Sonal Singh, Yoon K Loke

**Affiliations:** 1 Department of Medicine, Johns Hopkins University School of Medicine, Baltimore, USA; 2 Norwich Medical School, University of East Anglia, Norwich, UK

## Abstract

Randomized controlled trials are the principal means of establishing the efficacy of drugs. However pre-marketing trials are limited in size and duration and exclude high-risk populations. They have limited statistical power to detect rare but potentially serious adverse events in real-world patients. We summarize the principal methodological challenges in the reporting, analysis and interpretation of safety data in clinical trials using recent examples from systematic reviews. These challenges include the lack of an evidentiary gold standard, the limited statistical power of randomized controlled trials and resulting type 2 error, the lack of adequate ascertainment of adverse events and limited generalizability of trials that exclude high risk patients. We discuss potential solutions to these challenges. Evaluation of drug safety requires careful examination of data from heterogeneous sources. Meta-analyses of drug safety should include appropriate statistical methods and assess the optimal information size to avoid type 2 errors. They should evaluate outcome reporting biases and missing data to ensure reliable and accurate interpretation of findings. Regulatory and academic partnerships should be fostered to provide an independent and transparent evaluation of drug safety.

## Review

### Background

Randomized controlled trials are primarily designed to provide reliable information on the efficacy of interventions [1]. They form the primary basis of regulatory approval for a drug in the US, which involves demonstrating evidence of efficacy and safety in two well-conducted studies. With rare exceptions, these are generally interpreted as statistically significant data from two randomized clinical trials. Several advances have been made in the approach to the conduct, analysis and interpretation of data from randomized controlled trials on efficacy outcomes [1].

Since trials are typically carried out to define therapeutic benefit for regulatory approval, safety receives less attention [2]. The role of drug safety regulation is to protect patients from rare, severe adverse reactions; most efforts are directed at early detection and prevention of serious events such as that seen with thalidomide. Post-marketing surveillance through spontaneous adverse event reporting systems are the mainstay of drug safety evaluation. Methodological issues around the analysis of safety data from clinical trials have received less attention.

Systematic reviews and meta-analyses of clinical trials have recently raised concerns about an increase in the risk of serious adverse cardiovascular outcomes associated with varenicline [2-4], an increased risk of mortality associated with the tiotropium Respimat inhaler, and adverse cardiovascular outcomes associated with inhaled anticholinergics (including the ipratropium and tiotropium inhaler) [5,6]. Similarly, increased risks of myocardial infarction associated with rosiglitazone [7-9] and congestive heart failure and fractures associated with the thiazolidinediones (rosiglitazone and pioglitazone) in clinical trials have resulted in regulatory warnings [10,11]. These findings have been widely debated with conflicting interpretation by the academic community, regulators and industry sponsors [12-14]. Regulators have emphasized the limitations to defining and measuring adverse outcomes in randomized controlled trials and have called for caution in drawing any robust conclusions [12]. The lack of access to individual participant data, the heterogeneous nature of safety data, and the statistical challenges of analyzing rare events make safety data from such meta-analyses difficult to analyze and interpret [12-16]*.* This review summarizes the principal methodological challenges in the reporting, analysis and interpretation of safety data in clinical trials. We discuss potential solutions to these challenges.

## Methodological challenges

There are several challenges to identifying reliable drug safety signals in clinical trials.

### Lack of an evidentiary gold standard

There is no universally acceptable gold standard for determining whether a drug safety signal represents a true risk versus a false-positive signal. While evidentiary standards for efficacy are well established by regulatory statutes, the evidentiary standards for ascertaining safety are heterogeneous and encompass various data sources and study designs. Under the FDA Amendment Act of 2007, the FDA may revise a drug label to include a warning about a clinically significant hazard when “*there is reasonable evidence of a causal association with a drug*” [17]. However, what level of evidence constitutes reasonable evidence for regulatory action is open to interpretation.

In a recent review of regulatory actions by the FDA in 2009 [18], approximately two thirds of major regulatory actions on drug safety, including boxed warnings, withdrawals and contraindications, relied on spontaneous case reports [19]. Safety data from meta-analyses of clinical trials have resulted in some warnings, such as the warning on the potential association between antidepressants and suicidality [20] and boxed warnings on the risk of myocardial ischemic events with rosiglitazone [8].

### Limited statistical power

The premarketing clinical trials required for approval of a drug primarily guard against type 1 error. RCTs are usually statistically underpowered to detect the specific harm either by recruitment of a low-risk population or low intensity of ascertainment of events. The lack of statistical significance should not be used as proof of clinical safety in an underpowered clinical trial. As an example, varenicline is an alpha 4 beta 2 agonist approved for short-term abstinence among smokers based on small short-term placebo-controlled trials of efficacy [3-5]. Despite the high prevalence of psychiatric comorbidity and cardiovascular disease among smokers, the development program for varenicline largely excluded such patients [3]. Only one RCT was conducted among patients with cardiovascular disease. A meta-analysis of 14 double-blind placebo-controlled randomized controlled trials reported a statistically significant increase in serious adverse cardiac events with varenicline [3]. Another intensive post-marketing cohort study also reported on the underlying mechanisms by which this cardiovascular hazard is mediated [5].

Similarly, the reliance on surrogate endpoints to facilitate earlier access to drugs for chronic diseases limits the size and duration of trials in which efficacy can be demonstrated or where risk might be detectable [21]. The efficacy of an antidiabetic drug on glycated hemoglobin can be adequately demonstrated in premarketing placebo controlled trials of modest size and duration [22]. Recent regulatory requirements stipulate that drugs approved for the treatment of type 2 diabetes should not demonstrate a significant cardiovascular hazard (major adverse cardiovascular events) in preapproval trials with an upper bound of confidence interval of 1.8 [22]. There are no regulatory requirements that drugs approved for type 2 diabetes lower the micro- or macrovascular complications of type 2 diabetes [21]. Although a reliable diagnostic and prognostic surrogate, it is unclear whether glycated hemoglobin is a reliable treatment surrogate for drug approval of type 2 diabetes [21]. The linkage between glycated hemoglobin reduction and improved microvascular outcomes may be reasonable for metformin and sulfonylurea [23]. However, three large long-term trials have failed to show a cardiovascular benefit of intensive glycemic control [24-26], and one large trial reported an increase in mortality with intensive glycemic control [25].

### Lack of adequate ascertainment and classification of adverse events

The inconsistencies in adverse effects reported in clinical trials can create challenges. Adverse events are recorded as secondary outcomes in trials and are usually not prespecified. Misclassification of adverse events is possible, particularly where the outcomes are collected through spontaneous reports from trial participants rather than systematic monitoring. As an example, several recent systematic reviews have raised the possibility that the use of inhaled corticosteroids in patients with chronic obstructive pulmonary disease or thiazolidinediones among patients with type 2 diabetes may increase the risk of pneumonia [27-29]. Radiographic or microbiologic confirmation of pneumonia was not available. Pneumonia was not prespecified as an outcome of interest, but ascertained as adverse events or serious adverse events in these trials. Whether the risk of pneumonia seen in clinical trials of inhaled corticosteroids [27,28] represents a potential misclassification of a subset of COPD exacerbations is unknown. Similarly, the misclassification of congestive heart failure known to be associated with thiazolidinedione use may have resulted in the higher rates of pneumonia reported with thiazolidinediones in clinical trials [29].

### Limited generalizability

The lack of generalizability of certain RCTs needs to be acknowledged. Study participants are often carefully selected, and the trial may have been designed to evaluate only one particular dose, so there is no information on dose-responsiveness. Hence, it may be difficult to extrapolate the safety data to wider populations who may be taking different doses or formulations. As an example, the apparent safety of omeprazole when used together in a trial with clopidogrel has been questioned because of the proprietary formulation used [30].

In another example, a meta-analysis of 17 clinical trials reported a statistically significant increased risk of MI, stroke and CV death with inhaled anticholinergics (ipratropium bromide and tiotropium bromide) [7]. Data from another formulation of tiotropium bromide delivered via the Respimat inhaler showed a statistically significant increased risk of mortality [6]. Most of these deaths were cardiovascular deaths. These cardiovascular deaths were concentrated among patients with pre-existing arrhythmias and cardiovascular disease. There is biologic and clinical evidence that the cardiovascular risk of inhaled anticholinergics is particularly concentrated among patients with chronic obstructive pulmonary disease who also have concomitant cardiovascular disease [31]. Although a subsequent trial reported an increased risk of angina and arrhythmias [32], it reported no increased risk of myocardial infarction and composite stroke [32]. However, the exclusion of patients with cardiovascular disease limited the generalizability of its safety findings.

### Methodological opportunities

The analysis of safety data from clinical trials offers unique methodological opportunities. In the case of gastrointestinal hemorrhage with aspirin, even when the causal link between the drug and adverse effect is already well recognized, the data from relevant trials potentially allow greater precision in estimating the risk of harm [33]. This is particularly important where the benefit:harm ratio is finely poised or for drugs used in primary prevention where otherwise healthy patients have to live with adverse effects of treatment in the hope of avoiding some future event.

### Systematic assessment of different data sources

The adverse events vary from mild symptomatic events with a high background incidence to rare life-threatening conditions. The timing of the adverse effects in relation to duration of intervention may vary from immediate hypersensitivity reactions to long-term cancer risk many years down the line. Each source of data, be it spontaneous reporting or RCT, has its own strengths and limitations. The strengths and limitations of various data sources in assessing adverse effects are shown in Table 1.

**Table 1:** Key features and limitations of different approaches to assessing adverse effects of healthcare interventions

**Study design**	**Key features**	**Limitations**
Spontaneous or voluntary reporting systems, including journal-published case reports	Captures very wide range of events	No denominator or control group, difficult to quantify risk
	Particularly useful for detecting signals of rare (low background incidence in treated population) and/or unexpected events (e.g., new unrecognized pathology)	Format and type of information differs substantially among regulators and journals
	Sophisticated statistical techniques have been developed for signal detection	Clinical details may be incomplete, causality uncertain
		Selective reporting or under-reporting of cases
		
		
		
Randomized clinical trials	Randomization reduces possibility of confounding at baseline	Rigid recruitment criteria may lead to exclusion of patients who are at risk of adverse effects
	Certain adverse effects can be prospectively specified for detailed monitoring	Powered for detection of significant difference between groups for beneficial effect, estimates for adverse effects may lack precisions
	Intervention is typically well defined	
Non-randomized studies	‘Real-world’ use with more generalizable data and longer follow-up	Monitoring for rare or unexpected events may be less rigorous, and the trials may not be of sufficient duration to detect long-term problems
	Potentially able to specify and assess rare events as primary outcomes in case control designs	Non-randomized nature is susceptible to confounding
	May be able to explore relationship to dose, duration and patient susceptibility factors	Drug exposures are often based on computerized records rather than dispensing or actual use
Meta-analysis of controlled observational studies and/or trials	Pooled analysis has greater power to detect significant differences, even with rare events	Reliant on quality of primary data
		Missing or unreported data on adverse events is a major problem, as are the statistical techniques of pooling sparse data
	Aims to summarize complete data set and can evaluate consistency of findings among studies	Susceptible to selective outcome reporting of primary studies Heterogeneity within the pooled analysis

Appropriate data sources should be carefully considered and chosen depending on the adverse effect of interest and the study question. A single study design is unlikely to reliably measure all the different possible adverse effects, and focusing on certain study designs may prove more fruitful, depending on the background incidence of the particular outcome and the timing in relation to the intervention [33-35].

A comprehensive evaluation of the safety profile of a drug requires collecting and synthesizing data on a diverse range of adverse events [34,35]. The choice of appropriate study design is important not only because of differences in the nature of adverse effects, but also because the diversity and complexities of the safety questions can only be addressed through evaluating multiple sources of data [35]. A meta-analysis should ideally be preceded by the systematic review process where rigorous searches and assessment of validity and heterogeneity are carried out on the relevant trials to build a more complete data set rather than just considering single trials in isolation.

The adverse effects data from trials do not systematically differ from other study designs [36]. A recent study found that among 58 meta-analyses that provided comparative data on odds ratios from observational studies and trials, 93% had overlapping 95% confidence intervals [36]. There were no consistent differences between adverse effects data from RCTs compared to those of observational studies, with minimal differences in risk estimates from RCTs and symmetrical funnel plots.

Randomized controlled trials are suited for evaluating outcomes that occur fairly close to initiation of therapy and that have a relatively high baseline incidence even in untreated populations, and they provide the absolute or relative risk increase for a specific adverse event. The RCT generally offers an unconfounded comparison between groups of patients, where the only difference is the intervention delivered. The process of randomization with adequate allocation concealment guards against selection biases. Although randomized treat assignment in clinical trials reduces the possibility of confounding at baseline, it is possible that confounding may arise later from differential dropouts, crossover or changes in therapy.

In patients with type 2 diabetes mellitus, observational studies of cardiovascular harm with thiazolidinedione therapy as compared to metformin are potentially confounded by the clinical tendency to use metformin early on, while reserving thiazolidinediones for patients with poorly controlled diabetes. Thus, data from placebo-controlled RCTs have been critical in the evaluation of heart failure and risk of myocardial infarction with rosiglitazone [7-9]. In contrast, pioglitazone and rosiglitazone are second-line agents for the treatment of type 2 diabetes. Observational studies comparing the two thiazolidinediones are less susceptible to confounding by indication, because there are no apparent clinical reasons why prescribers would selectively channel patients to either rosiglitazone or pioglitazone [10].

The strength of RCTs lies in the potential to maintain blinded conditions when assessing adverse effects. The use of placebo reduces the tendency for bias in selectively reporting or diagnosing adverse events. Lack of blinding is particularly relevant when considering subjective symptoms where participants’ inclination to report certain events may be swayed by anticipation of adverse affects potentially associated with the drug. Equally, physicians may have a greater tendency to investigate, diagnose and report specific adverse events that they consider likely to be related to the particular drug intervention.

Since the analytical strength of the RCT lies in the comparison between groups, these may not be the most appropriate design for signal detection of rare, unexpected effects or for directly unveiling susceptibility factors in subgroups of participants. However, the availability of individual patient data may subsequently make this possible. Data from spontaneous reporting systems may be of particular interest for detecting signals for rare, unusual adverse events, such as the risk of suicide with varenicline [19]. However, they have their own limitations, including the lack of an appropriate denominator [37]. In certain instances even spontaneous reports may result in decisive regulatory action [37]. After the approval of felbamate for epilepsy, there were spontaneous reports of aplastic anemia at rates several times than that of historical controls. This resulted in its withdrawal from the market.

### Statistical analysis

Attention to the intention-to-treat analysis of all randomized participants is important. Conducting the analysis using time on treatment or based on the extent of drug exposure may result in loss of the benefit of randomization and creation of a confounded comparison. However, in the case of a non-inferiority open label safety trial, the conduct of an ITT analysis may provide false reassurance of safety. In the Rosiglitazone Evaluated for Cardiac Outcomes in Dysglycemia (RECORD) Trial, the ITT analysis did not demonstrate non-inferiority on cardiovascular outcomes with rosiglitazone versus the comparator [38]. However, in non-inferiority trials both ITT and on-treatment analyses need to be examined before ruling out the presence of a significant safety hazard [39].

The appropriate statistical analysis plan should also consider the biological plausibility and latency of the adverse effect. The evaluation of the possibility of an increased cancer risk with drugs should consider the latency period and avoid lumping together short-term trials (e.g. of less than a few months duration, where no such risk can be detected) with long-terms studies, which may lead to the dilution of the cancer risk. However, too long a latency period should be avoided because often the specific timing of the hazard is unknown.

As an example, in the PROspective pioglitAzone Clinical Trial In macroVascular Events (PROactive) trial, there were 20 reported bladder tumor cases: 14 in the pioglitazone group (*n* = 2,605) and 6 in controls (*n* = 2,633) [40]. An initial assessment by the study investigators concluded that 11 tumors that occurred within 1 year of randomization (eight pioglitazone, three placebos) could not plausibly be related to treatment. If pioglitazone accentuates the risk of bladder cancer in patients with type 2 diabetes, then 1 year may be a reasonable time frame, and such cases of bladder cancer should not be removed from the statistical analysis. After removing one case of bladder tumor in the placebo arm that was benign, there was a statistically significant increased risk of bladder cancer with pioglitazone in the PROactive trial: 0.54% (14/2,605) cases of bladder cancer in the pioglitazone arm versus 0.19% (5/2,633) cases of bladder cancer in the control arm, respectively (RR 2.83; 95% CI 1.02—7.85, *P* = 0.040) [41]. Pioglitazone was known to be associated with bladder cancer in animal studies prior to regulatory approval [41]. Although future observational studies have provided further confirmation on the strength of evidence of this association, conclusive proof linking pioglitazone to bladder cancer in clinical trials was readily available in 2005, nearly half a decade before clinicians and patients were warned of such risks.

### Ascertainment of adverse events

New or unexpected adverse effects cannot be prespecified in trial protocols. Even if such adverse effects were not primary or secondary outcomes, any substantial imbalance in rates between intervention arms in a trial should be evaluated. Any weaknesses in ascertainment in a randomized, double-blinded trial that should affect the intervention arms equally are likely to be non-differential and bias estimates toward null. A consistent increase in the risk of pneumonia reported as an adverse event or a serious adverse event in clinical trials of inhaled corticosteroids increases our confidence in the strength of this association [27]. Potential misclassification of heart failure associated with thiazolidinediones as pneumonia was a concern in clinical trials of the thiazolidinediones, which reported an increased risk of pneumonia. However, readjudication of heart failure in the PROactive trial showed that the impact of such misclassification was likely minimal [29].

To avoid ascertainment biases, trial protocols should pre-specify monitoring for prespecified adverse events based on pharmacological mechanisms or data from earlier studies. Following publication of initial case reports of newly diagnosed congestive heart failure with thiazolidinediones, subsequent RCTs were able to report heart failure events with much greater detail, including adjudication by independent committees [26]. The true role of adjudication of safety events in clinical trials remains unclear [42]. The reliance only on adjudicated major events may further reduce statistical power because of a lower number of adverse events recorded. Thus, sensitivity analysis using both adjudicated as well as all serious adverse events should be considered [3]. However, if the trial was appropriately randomized and blinded, the presence or absence of adjudication should not differentially affect the relative rates of adverse events between comparator arms. Reviewers and trialists should attempt to measure blinding failure and blinding biases when possible.

### Statistical modeling of rare events in meta-analysis

The exact choice of statistical methods to evaluate safety data will depend on the individual context. There is general consensus that safety data should be modeled on the relative scale as absolute risk models are underpowered and result in type 2 error. The assessment of statistical heterogeneity is appropriate but of lesser concern when dealing with rare but serious adverse events where the primary focus is on detecting a signal if it exists. The commonly employed tests for statistical heterogeneity, e.g., Cochran’s test, are relatively underpowered. The Peto odds ratio (OR) method with 95% confidence intervals may provide the best confidence interval coverage, and is more powerful and relatively less biased than random effects analysis when dealing with low event rates [15]. Sensitivity analyses using alternative statistical approaches such as the fixed Mantel-Haenzsel odds ratio can also explore the influence on effect size of the reciprocal of the treatment arm continuity correction or no continuity correction for trials with zero events [3,16].

### Overcoming outcome reporting and funding biases and missing data

Outcome reporting bias has also been identified as a major issue in the assessment of efficacy and safety data in industry-sponsored trials [43]. Reviewers should attempt to identify data from multiple sources including clinical trials.gov and regulatory documents as sponsors may omit such key safety information from published trials, as demonstrated in the case of pioglitazone above. The availability of complete adverse effects reports from the sponsor’s register has enabled far more detailed analysis of cardiovascular events with rosiglitazone than pioglitazone [7]. While pharmaceutical support does not automatically make an analysis unreliable, the potential biases of industry-sponsored investigators providing proof of safety deserve closer scrutiny. In the case of rosiglitazone, an analysis revealed that the investigators position on the cardiovascular risks of rosiglitazone was closely aligned with their sources of pharmaceutical support [44].

Loss to follow-up, particularly in long-term trials, is a problem, particularly where there are differential losses between intervention arms. The benefits of randomization will be diminished if the patients who continue within a particular intervention arm are different from those originally randomized, thus leading to potential confounding. Equally, the presence of differential losses and the cessation of adverse event monitoring in patients who have withdrawn could lead to imbalances in the rates of adverse effects between arms. Distinction should be made between patients who have completely left the trial as opposed to those who have stopped taking the trial drug but remain available for follow-up of adverse events. This should be built into the original submission for ethical approval, and patients re-consented at the time of stopping trial treatment. This does not eliminate the possibility of confounding but reduces the problem of missing adverse effects data for patients who have withdrawn. Trials need to collect safety data on prematurely withdrawn participants, and safety data from such participants should be included in the analysis. It was only after the inclusion of mortality data on prematurely censored participants that a statistically significant increased risk of mortality with tiotropium Respimat in patients with COPD was demonstrated in clinical trials [6].

### Interpretation of safety data from trials

One needs to carefully consider the limitations of the sample size and duration of the intervention in the context of the adverse effect where ‘no significant harm’ is reported. Undue reliance on the thresholds of statistical significance or the small magnitude of statistical effect should be avoided. A mere 8% increase in the risk of diabetes with statin therapy has potential public health significance, irrespective of the threshold of statistical significance [45]. The width of the 95% confidence intervals, including the upper bounds, provides better assessment of a clinically significant hazard in the context of the benefit.

The power of the meta-analysis depends on the number of events, not the number of trials. Underpowered statistical models that utilize the absolute risk scale or prematurely censor participants who remain in the trial may further reduce the power of a meta-analysis. Such underpowered safety studies should be interpreted with caution. A meta-analysis that provides proof of safety should be accompanied by information on the optimal information size [46]. Evaluating the upper bounds of the 95% confidence intervals is important when the purpose is to rule out a significant risk

## Conclusions

Trials are usually powered to detect benefit and seldom designed with adverse events as primary outcome. It is not possible to design trials to evaluate unexpected or unknown adverse effects that have yet to be linked to the intervention. Clinical trials should include explicit pre-specified monitoring of pharmacologically predictable adverse events and ensure adequate follow-up of withdrawn participants. Recent regulatory guidance from the FDA has limited the reporting of adverse events in clinical trials from sponsors to those that are unexpected and considered related to the drug [47]. It is unclear how isolated investigators will determine the causal relationship between a drug and its adverse events. The expanding role of electronic trials registers with detailed study results has potential that can only be fully realized when sponsors provide reliable, accurate and complete data [48].

Empirical work is needed to evaluate whether novel approaches such as mechanism-based drug toxicity prediction can complement safety data from clinical trials and improve an assessment of drug safety [49]. Methodological research is needed to determine whether network meta-analysis techniques can provide reliable and valid comparative evaluation of drug safety [50]. The European Medicines Agency has recently undertaken methodological work to enhance the consistency and transparency of their risk-benefit decision-making process [51]. Current proposals emphasize the need to not just consider the magnitude and consequences of treatment effects, but also to evaluate less tangible factors such as the level of uncertainty and extent of risk tolerance. These developments are particularly relevant when considering rare but serious adverse events where the clinical trials may yield imprecise or even conflicting estimates. Multi-criteria decision analytical techniques that accurately capture quantitative inputs and qualitative values from various stakeholders for risk-benefit tradeoffs and allow for quantitative analysis and modeling uncertainty on a range of outcomes can improve complex regulatory decisions about drug safety.

Conducting health outcome trials prior to approval increases the evidence base on safety. Post-marketing safety studies of adequate design become mandatory in circumstances when surrogate endpoints are used to approve a drug. Regulators should be provided with adequate resources and expertise to conduct such safety evaluations. Regulatory and academic partnerships should be fostered to provide an independent and transparent evaluation of drug safety. The increasing pressure by the public to make all clinical trial results available and heightened public awareness about emerging drug safety issues ensure that analyses of safety data from clinical trials will remain central to the discussion around drug safety in the foreseeable future.

## Competing interests

The authors declare that they have no competing interests.

## Authors’ contributions

SS and YKL helped to conceptualize the article, interpret findings and review drafts of the article. Both authors read and approved the final manuscript.
